# Association between whole-grain intake and myopia in chinese children: a cross-sectional epidemiological study

**DOI:** 10.1186/s12886-022-02764-6

**Published:** 2023-01-02

**Authors:** Zhuzhu Liu, Qingxin Wang, Qianyu Zhao, Fei Gao, Nan Jin, Di Wang, Biying Wang, Bei Du, Ruihua Wei

**Affiliations:** 1 Tianjin Key Laboratory of Retinal Functions and Diseases, Tianjin Branch of National Clinical Research Center for Ocular Disease, Eye Institute, School of Optometry, Tianjin Medical University Eye Hospital, Tianjin, China; 2 State Key Laboratory of Experimental Hematology, National Clinical Research Center for Blood Diseases, Institute of Hematology & Blood Diseases Hospital, Chinese Academy of Medical Sciences & Peking Union Medical College, Tianjin, China

**Keywords:** Myopia, Protective factors, Diet, Whole grains, Near work

## Abstract

**Background:**

Nutritional status influences the growth and development of the eyes. However, there are few studies on the association between diet, especially whole grains (WG) consumption, and myopia. The study aimed to evaluate the association between WG intake and myopia prevalence among primary school-age children in China.

**Methods:**

This cross-sectional epidemiological study conducted between November 2019 and December 2019 included 586 children, aged 6–12 years, attending primary school in Binhai district, Tianjin, China. Ophthalmologic examinations and optometric cycloplegic refraction measurements were conducted. Information was collected on known risks and protective factors for myopia and the consumption of WGs, vegetables, and fruits. This association between the probability of myopia and the proportion of WG consumption (WG proportion was calculated as the mean intake from WG sources divided by total grain intake), adjusted for protective and risk factors, was analysed using crude and multivariable logistic regression.

**Results:**

Among the study participants, 226/586 (38.57%) children had myopia in at least one eye. WG intake was inversely correlated with the prevalence of myopia. Furthermore, in the multivariate analysis, WG intake of > 50% was identified as a protective factor against myopia after subsequent adjustment for children’s age, sex, parental myopia, near-work activity, screen time, reading and writing habits, visual fatigue, outdoor time, and classroom light environment (all *P* < 0.05).

**Conclusion:**

WG intake (> 50%) was an independent protective factor against myopia. Modifying the form of grains consumed (whole versus refined) could be one of the targets of future public health measures.

## Background

Myopia is a significant public health problem worldwide and the largest attributable risk factor for vision loss. It has been predicted that 4.8 billion people (approximately 60% of the world’s population) will exhibit myopia worldwide by 2050 [1]. Myopia is an irreversible eye disease, often accompanied by complications, such as retinal detachment, macular disease, macular haemorrhage, choroidal neovascularisation, and glaucoma, when it progresses to high myopia [2]. Therefore, it is necessary to investigate the factors influencing this rapidly growing prevalence.

With the rapid time-scale increase in myopia (less than 2 to 3 generations), several non-genetic associated factors have been identified. Time spent doing near-work, screen time duration, and less time outdoors are the most frequently cited environmental factors underlying the development of myopia [3–6]. During the developmental ages at which myopia develops in children, diet, as a non-genetic associated factor, is primarily set by family characteristics, including wealth and culture. Economic development is often accompanied by diet changes, as reflected in the secular increases in height reported in many countries worldwide. Axial length growth is driven by physical development at an early age, whereas its excessive growth is dominated by myopia development [7]; thus, it is plausible that deficits in nutritional intake and status could play a role in its development and progression. To date, the association between diet and myopia has not been extensively studied, especially in Chinese children. Over 200 years since the Industrial Revolution, a change has occurred from low to high-glycaemic load carbohydrates, as increasingly higher levels of refined sugars have been incorporated into the diet along with more refined grains [8]. In 2002, Cordain et al. [9] proposed that consumption of refined carbohydrates (starches and sugars) could be involved in the development of juvenile-onset myopia via hyperinsulinism; increased insulin may affect the hormonal regulation of eye growth and increase axial elongation. This hypothesis has been supported by more recent evidence [10–15]. Grain products, especially those made from whole grains (WGs), have received particular interest because they can influence the digestion rate and decrease the blood glucose response. Studies on WGs intake have reported a reduced risk of type II diabetes with higher consumption of WGs [16, 17]. Moreover, WGs constitute a significant source of beneficial nutrients, such as dietary fibre, phytochemicals, minerals, several vitamins, and other supplements [18, 19]. All these ingredients are abundant in WGs but are lacking in refined grains, which are a major source of high-glycaemic carbohydrates.

Currently, the long-term effect of WG versus refined grain consumption on the prevalence of myopia has not been extensively studied, especially in Chinese children whose lifestyle and diet patterns have changed dramatically. This study aimed to evaluate the association between WGs and the prevalence of myopia to provide comprehensive evidence to improve prevention, treatment, and management of myopia.

## Methods

### Study design and population

A multi-stage stratified cluster sampling method was adopted, and three classes were randomly selected in each grade (1–6). Ultimately, 634 students in 20 classes were included in this study. The inclusion criterion was children aged between 6 and 12 years. The exclusion criteria were (1) a history of systemic or ocular disease that required treatment, including keratitis, ocular allergic disease, any other ocular surface disease, glaucoma, active or chronic uveitis, or previous ocular surgery or injury, and (2) a history of using atropine eyedrops, orthokeratology, or any method to prevent or control myopia.

The study protocol was approved by the Institutional Ethics Committee of Tianjin Medical University Eye Hospital (approval no. 2019KY-12) and followed the tenets of the Declaration of Helsinki for research involving humans. The parents or legal guardians provided written informed consent on behalf of all participants.

### Eye examination and measurement

All children underwent comprehensive eye examinations, including measurements of distance visual acuity and intraocular pressure, non-cycloplegic and cycloplegic refraction, slit lamp microscopy, and digital fundus photography. The international standard visual chart was used to evaluate uncorrected and corrected visual acuity. A slit lamp, tonometer, and ophthalmoscope were used for ocular health examination. In addition, cycloplegia was achieved by instilling three drops of 1% cyclopentolate 5 min apart in each eye. Subsequently, 30 min after the last drop, another drop of 1% cyclopentolate was administered if the pupil size was < 6 mm or if the pupillary light reflex was still present. Autorefraction was performed 35 min after the last drop.

Refraction was measured in patients with ciliary paralysis using an Auto Refractometer (KR-800, Topcon, Tokyo, Japan), and three successive measurements were recorded and averaged. All three readings had to be ≤ 0.25 D apart in both the spherical and cylindrical components. The spherical equivalent refraction was calculated as the spherical refractive error + 1/2 of the cylindrical refractive error, and participants with a spherical equivalent refraction ≤ − 0.50 D in either eye were classified as having myopia.

### Dietary intake assessment

Food consumption data were collected through a dietary history interview, including the habitual diet over the past year. The interviewers were nutritionists who used a questionnaire listing 122 foods, with the usual serving sizes of WGs, refined grains, vegetables, and fruit items in common household measures (e.g., soup spoons, rice bowls, and cups). The children’s parents or guardians were asked to recall the usual intake of each food item on the list for the past year as an indicator of the participants’ long-term diet. Food images were provided, and food models were used to help participants remember the portion sizes of specific foods.

WG variables were classified using a modified version of Jacobs et al.‘s procedure [20]. Individual WG foods (serving/day), including WG flour and other products (Coix seed, brown rice, sorghum, black rice, barley, yellow rice, corn, oats, highland barley, buckwheat, wheat, millet, and amaranth grain), with a WG or bran content ≥ 25% by dry weight, were calculated by multiplying the pre-specified serving size by the reported intake frequency of each food. Fruit intake was calculated by summing the servings of strawberries, prunes, bananas, blueberries, grapefruit, cantaloupe, raisins, apples, blueberries, pears, peaches, watermelon, and oranges. Vegetable intake was estimated by summing the servings of alfalfa sprouts, cabbage, coleslaw, cauliflower, Brussels sprouts, carrots, corn, peas, mixed vegetables, beans, broccoli, lentils, yams, spinach, squash, kale, lettuce, beets, celery, tomatoes, and potatoes. Fruit and vegetable juices were not included in the assessment of fruit and vegetable intake. The ingredients of the mixed foods were broken down into their component foods and assessed using a recipe file. Finally, the reported portion of each serving was converted to gram weight, and the intake of different food items was combined.

### Population categorisation

The proportion of WG consumption was calculated as the mean intake from WG sources divided by total grain intake. The parents or guardians self-reported the proportion of WG in all grains, fruit intake (g/day), and vegetable intake (g/day). Furthermore, participants were categorised into three WG intake groups based on the proportion of WG in all grains consumed per day: low WG (< 25%), medium (25–50%), and high (> 50%). This categorisation was chosen following the dietary guidelines of the United States, which recommend that consumers eat at least half their grains as WGs [21]. Moreover, the study population was divided into tertiles based on total dietary vegetable and fruit intake (g/day): vegetable (< 75 g/day, 75–150 g/day, > 150 g /day) and fruit (< 75 g/day, 75–150 g/day, > 150 g/day). All tests were performed with the proportion of WG in all grains as the exposure measure, and one-day recall data were estimated using a predictor of true nutrient intake, including fruits and vegetables as covariates, to aid estimation.

### Covariate measurements

Participants completed questionnaires to provide information on various known or suspected risks and protective factors for myopia as follows: age; sex; parental myopia (including the number of parents with myopia and high myopia); near-work activity (including distance and hours per week); screen time (hours per week using electronic devices and watching television); reading and writing habits (including reading posture, the distance between fingertip and nib, and head angle); frequency of visual fatigue per week; outdoor times; and light environment in the classroom. These factors were included as covariates in different multivariable logistic regression models, as these elements have been demonstrated to have possible associations with myopia in previous studies [5, 22–25].

### Statistical analysis

Descriptive results for the participant characteristics are described using means (standard errors) for continuous variables and frequencies (percentages) for categorical variables. Participant characteristics were compared between the non-myopia and myopia groups using Wilcoxon rank sum tests, Z statistic for continuous variables, and the χ^2^ test for categorical variables. To estimate the relationship between WG and the health outcome of myopia, univariate and multivariate logistic regression models were constructed to determine the relationship between WGs and the prevalence of myopia. Usual WG intake was treated as an explanatory variable for myopia in univariate and multivariate logistic regression, with < 25% as the reference category.

Model 1 was adjusted for age, sex, and school type (public versus private), while Model 2 was additionally adjusted for parental myopia. In Model 3, covariates, including near-work activity, screen time, reading and writing habits, and times of visual fatigue per week, were further added to the model, Model 4 was additionally adjusted for time spent outdoors per week and light environment in the classroom (whether classroom curtains are often open). Model 5 was further adjusted for intake of fruits and vegetables. The effect modification of associations between WG and myopia incidence was tested by stratified analyses of age (6–8 years vs. 9–12 years) and sex (female vs. male). P-interaction values were calculated using the likelihood ratio test to include a cross-product term of the ordinal categorical exposure and a 2-category effect modifier. All probabilities quoted were two-sided, and all statistical analyses were performed using the Statistical Package for Social Sciences version 13.0 (SPSS Inc., Chicago, IL, USA). A *P* value ≤ 0.05 was considered significant.

## Results

A total of 586 students (273 girls and 313 boys) completed the examinations and questionnaire survey between November 2019 and December 2019, and the response rate was 92.43% (586/634). Of the 586 participants, a significant proportion (38.57%) had myopia, and the average equivalent spherical power of 1172 eyes was − 0.11 D ± 1.63 D. The characteristics of the patients with and without myopia are shown in Table 1.

**Table 1:** Characteristics of all participants

Characteristics	All participants (586)	Subgroup participants				*P* value
		Non-myopia (360)		Myopia (226)		
**Age**						*P* < 0.001**
6–8 years	333	268 (80.48%)		65 (19.52%)		
9–12 years	253	92 (36.36%)		161 (63.64%)		
**Sex**						*P* = 0.001**
Female	273	148	(54.21%)	125	(45.79%)	
Male	313	212	(67.73%)	101	(32.27%)	
**Duration of electronic device use per week**						*P* = 0.078
0–7 h	328	213	(64.94%)	115	(35.06%)	
7–14 h	184	110	(59.78%)	74	(40.22%)	
14–21 h	50	27	(54.00%)	23	(46.00%)	
>21 h	24	10	(41.67%)	14	(58.33%)	
**Duration of watching television per week**						*P* = 0.594
0–7 h	416	250	(60.10%)	166	(39.90%)	
7–14 h	126	84	(66.67%)	42	(33.33%)	
14–21 h	30	18	(60.00%)	12	(40.00%)	
>21 h	14	8	(57.14%)	6	(42.86%)	
**Duration of near work per week**						*P* < 0.001**
0–25 h	189	136	(71.96%)	53	(28.04%)	
25–38 h	311	182	(58.52%)	129	(41.48%)	
>38 h	86	42	(48.84%)	44	(51.16%)	
**Near-work distance**						***P*** = 0.017**
>33 cm	283	188	(66.43%)	95	(33.57%)	
<33 cm	303	172	(56.77%)	131	(43.23%)	
**Times of eye exercise per week**						*P* = 0.280
1–5 times	285	183	(64.21%)	102	(35.79%)	
5–10 times	246	140	(56.91%)	106	(43.09%)	
10–20 times	52	35	(67.31%)	17	(32.69%)	
>20 times	3	2	(66.67%)	1	(33.33%)	
**Times of visual fatigue per week**						*P* = 0.021*
0 time	338	221	(65.38%)	117	(34.62%)	
1–5 times	231	134	(58.01%)	97	(41.99%)	
5–10 times	12	4	(33.33%)	8	(66.67%)	
> 10 times	5	1	(20.00%)	4	(80.00%)	
**Reading posture**						*P* = 0.022*
Sitting	365	235	(64.38%)	130	(35.62%)	
Prone	175	93	(53.14%)	82	(46.86%)	
Supine	46	32	(69.57%)	14	(30.43%)	
**Distance between fingertip and nib**						*P* = 0.082
<1.5 cm	218	124	(56.88%)	94	(43.12%)	
>1.5 cm	368	236	(64.13%)	132	(35.87%)	
**Head angle**						*P* = 0.233
Vertical 0°	44	26	(59.09%)	18	(40.91%)	
Left 0°–45°	217	128	(58.99%)	89	(41.01%)	
Left 45°–90°	250	165	(66.00%)	85	(34.00%)	
Right 0°–45°	57	30	(52.63%)	27	(47.37%)	
Right 45°–90°	18	9	(50%)	9	(50%)	
**Light environment in classroom**						*P* < 0.001**
Closed curtains	92	39	(42.39%)	53	(57.61%)	
Open curtains	516	335	(64.92%)	181	(35.08%)	
**Outdoor time per week**						*P* = 0.026*
0–7 h	268	150	(55.97%)	118	(44.03%)	
7–14 h	257	166	(64.59%)	91	(35.41%)	
>14 h	61	44	(72.13%)	17	(27.87%)	
**Parents with myopia**						*P* = 0.002*
0	133	96	(72.18%)	37	(27.82%)	
1	253	158	(62.45%)	95	(37.55%)	
2	200	106	(53.00%)	94	(47.00%)	
**Parents with high myopia**						*P* = 0.532
0	422	257	(60.90%)	165	(39.10%)	
1	130	79	(60.77%)	51	(39.23%)	
2	34	24	(70.59%)	10	(29.41%)	
**Vegetable intake per day**						*P* = 0.852
< 75 g	179	113	(63.13%)	66	(36.87%)	
75–150 g	312	189	(60.58%)	123	(39.42%)	
> 150 g	95	58	(61.05%)	37	(38.95%)	
**Fruit intake per day**						*P* = 0.545
<75 g	295	177	(60.00%)	118	(40.00%)	
75–150 g	244	156	(63.93%)	88	(36.07%)	
>150 g	47	27	(57.45%)	20	(42.55%)	
**Percentage of grains in staple foods**						*P* = 0.052
<25%	362	213	(58.84%)	149	(41.16%)	
25–50%	179	112	(62.57%)	67	(37.43%)	
>50%	45	35	(77.78%)	10	(22.22%)	
**School type**						*P *= 0.107
Public schools	560	348	(62.14%)	212	(37.86%)	
Private schools	26	12	(46.15%)	14	(53.85%)	

*, *P* < 0.05 is significant; **, *P* < 0.001 is highly significant

**Fig. 1:**
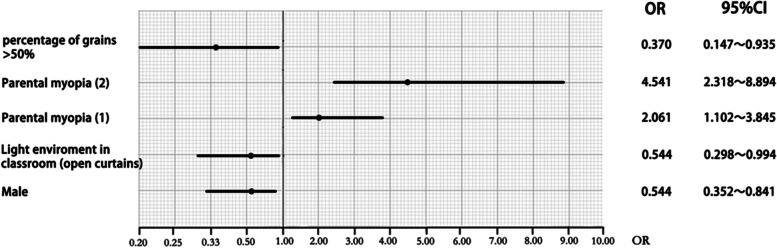
Forest plot showing the risk/protective factors for myopia in multivariate logistic analysis

Significant differences were observed regarding age, sex, parental myopia, near-work activity (distance and hours per week), reading posture, times of visual fatigue per week, the light environment in the classroom, and outdoor time between participants with and without myopia (*P* < 0.05). Different multivariable logistic regression models and variance inflation factors (AIC) were checked to avoid over-correcting. The VIF of all variables were below 10, indicating no multicollinearity problems. In multivariate logistic analysis, compared with having parents without myopia, having more parents with myopia (one parent with myopia, odds ratio [OR] = 2.061, *P* = 0.024; two parents with myopia, OR = 4.541, *P* < 0.001) was positively associated with myopia, but male sex (OR = 0.544, *P* = 0.006), elevated light levels in the classroom (classroom curtains are often open, OR = 0.544, *P* = 0.048), and higher proportions of WGs (> 50%, OR = 0.370, *P* = 0.036) were negatively associated with myopia (Fig. 1).

The association between WG intake and myopia in crude and multivariable logistic regression models is presented in Table 2. In the crude model, we used the presence of myopia as a dependent parameter and a WG percentage ≤ 25% as the reference. A 25–50% WG intake was not a significant factor in the prevalence of myopia (OR = 0.855, *P* = 0.405), but intake > 50% (OR = 0.48; *P* = 0.017) was a protective factor against myopia. Multivariable-adjusted ORs (95% confidence intervals) for myopia are shown in Models 1–5. In Model 1, using age, sex, and type of school (public versus private) as covariates, we found that the prevalence of myopia remained significantly associated with WG consumption > 50% but not with WG intake of 25–50% (*P* = 0.446). In Model 2 (parental myopia was additionally entered into the logistic regression model), > 50% WG intake remained significant. Further adjustments for near-work activity, screen time, reading and writing habits, and times of visual fatigue per week in Model 3 and light environment in Model 4 (outdoor times per week and light levels in the classroom) did not significantly alter the association. Additional adjustments for dietary factors in Model 5 (intake of fruits and vegetables) did not substantially alter the association. Conversely, fruit and vegetable intake was not significantly associated with myopia’s prevalence in the simple or multivariable logistic regression models (*P* > 0.05).

**Table 2:** Effect of whole-grain consumption on the odds of myopia in Models 1–5

	Whole grains: 25–50%	Whole grains: >50%
Crude model		
OR	1.033	0.376
95% CI	0.672–1.589	0.165–0.856
*P*-value	0.881	0.020
**Model 1**		
OR	1.033	0.376
95% CI	0.672–1.589	0.165–0.856
* P*-value	0.881	0.020
**Model 2**		
OR	1.160	0.381
95% CI	0.742–1.814	0.161–0.900
* P*-value	0.516	0.028
**Model 3**		
OR	1.221	0.398
95% CI	0.759–1.966	0.162–0.983
*P*-value	0.411	0.046
**Model 4**		
OR	1.252	0.393
95% CI	0.774–2.025	0.158–0.974
* P*-value	0.360	0.044
**Model 5**		
OR	1.217	0.370
95% CI	0.745–1.990	0.147–0.935
*P*-value	0.433	0.036

The reference category was WG intake < 25%

In the crude model: no adjustment, whole grains ≤ 25% were taken as reference. In Model 1, sex, age, and school type were adjusted. In Model 2, parental myopia was also included. In Model 3, near-work activity, reading and writing habits, screen time, eye exercise, and visual fatigue times per week were subsequently entered into the model. In Model 4, outdoor time and light environment in the classroom were further entered into the model. In Model 5, the intake of fruits and vegetables were also entered into the model

*CI *Confidence interval, *OR *Odds ratio

Results from the analyses stratified by sex and age are shown in Table 3. No interactions were observed for age (*P* = 0.846) and sex (*P* = 0.978) in the relationship between the intake of WG and myopia. When stratified by sex, a stronger association with WGs was observed among female students than male students. For boys, WG intake of 25–50% (*P* = 0.485) was not a protective factor; only an intake > 50% of WGs was identified as a protective factor against myopia (OR = 0.209, *P* = 0.047). In girls, when adjusting for covariates, WG consumption > 50% still significantly influenced the prevalence of myopia (OR = 0.214, *P* = 0.041). Furthermore, WG intake was non-significant in children aged 6–8 years, whereas WG intake > 50% remained protective against myopia for children aged 9–12 years.

**Table 3:** Effect of whole-grain intake on myopia stratified by sex and age (Adjusted OR and 95% confidence intervals)

	Proportion of whole-grain intake			*P*-trend
	< 25%	25–50%	> 50%	
Sex				
Female	1	1.663 (0.767–3.604)	0.214 (0.049–0.938) *	0.390
Male	1	0.739 (0.347–1.573)	0.209 (0.045–0.979) *	0.169
Age				
6–8 years	1	0.903 (0.429–1.898)	0.412 (0.113–1.494)	0.239
9–12 years	1	1.413 (0.663–3.010)	0.196 (0.044–0.872) *	0.115

The reference category was whole grain (WG) intake < 25%

Multivariable logistic regression was used to estimate the association between WG intake and incidence of myopia. When stratified by sex and age, all elements were adjusted in the models, except for themselves

*OR*  Odds ratio

* *P* <0.05

## Discussion

This study aimed to evaluate conjoint modifiable risk factors involved in myopia prevalence in a Chinese children population, including the impact of WG consumption. In this cross-sectional study, we found a significant association between increased WG intake and reduced myopia prevalence, especially among children aged 9–12 years, after adjusting for several demographic and lifestyle factors. Some risk/protective factors of myopia were concurrently detected: parental myopia was significantly associated with an increased probability of myopia, but male sex and elevated light environment in the classroom seemed protective.

Many studies have confirmed that a family history of myopia is a risk factor for developing myopia [26, 27], which was similarly observed in the present study. Multivariable logistic regression analysis showed that compared to children without a myopic parent, children with one myopic parent (OR = 2.061, *P* = 0.024) and both myopic parents (OR = 4.541, *P* < 0.001) were at a higher risk to prevalence of myopia. Josh Wallman pointed out that the effect could possibly as well be explained if myopic parents, who are more likely to be wealthier and better educated, create more myopigenic environments [28]. Recently, Guggenheim has also suggested that genetic effects explain only part of the impact of parental myopia [29]. Currently, hundreds of genetic loci have been identified for refractive error and myopia, and risk variants mostly carry low risk but are highly prevalent in the general population. Most researchers have now accepted the argument that genetics cannot have a major role, except in the rare cases that are predominantly genetic, because the rate at which gene pools change is inconsistent with the speed at which the epidemic of myopia has emerged [30, 31]. Like many other traits, common myopia is complex in its pathogenesis and progression, with contributions from genetic and environmental factors, including lifestyle, reduced outdoor time, and intensive near-work activities [5, 22–25]. A combination of various factors, rather than a single isolated factor, may determine the refractive state. In addition, we found that sex was associated with myopia, with reduced probability for boys (OR = 0.544, *P* = 0.006). This is likely because females spend more time studying, reading, and practising near-work and less time on outdoor activities at all ages [25, 32]. Among the associated factors for myopia in the multivariable logistic regression analysis, we were particularly surprised by the markedly lower prevalence of myopia in the participants where classroom curtains were often open. This would be consistent with the light effects reported by Cohen and Iribarren [33]. The link between light exposure and the prevalence of myopia has not been fully elucidated; however, the proposed reasons include factors such as variations in chromatic light composition, higher light intensities, and increased release of dopamine from the retina, which can slow axial elongation [34–38].

Gardiner [39] has suggested that accelerated growth patterns are associated with refractive errors in myopia, and that diet may be a potential environmental element common to both generalised accelerated growth and the onset of myopia progression. The results indicated that WG intake was an independent protective factor against myopia, and only those with > 50% WGs in their total grain-based food received significant benefits after adjusting for vegetable intake, fruit intake, and other covariates. Therefore, a beneficial link between WG intake and myopia can be attributed to specific factors associated with WGs.

Myopia is caused by environmental or genetic factors resulting in abnormal visual information acting on the retina, which is formed by retinal pigment epithelial–choroidal signal transduction. This ultimately acts on the sclera and initiates scleral remodelling. Dietary changes, including increased consumption of high-glycaemic carbohydrates, may also affect the structure of the growing eyes [40, 41] and lead to unregulated cell proliferation in scleral tissue [42]. WG foods are a healthier option than refined grains and include some parts of the grain that are removed during processing. WGs are nutritionally superior to refined grains because they are a major contributor of fibre; vitamins; minerals; zinc; and phytoestrogens, such as lignins [43]. Importantly, WGs contain higher amounts of dietary fibre than refined grains, and there is substantial collinearity between estimates of WG intake and cereal fibre intake [44]. Increased WG intake may release various dietary fibres that can slow the absorption and digestion of carbohydrates, reducing insulin demand [45]. An evolutionary analyses of the cause of myopia in adolescents by Cordain et al. [9] suggested that hyperinsulinemia diets promote myopic axial elongation by increasing insulin-like growth factor-1 circulation, reducing growth hormone circulation, and contributing to scleral growth through vitamin A and retinoid receptor signalling. Evidence from animal models also supports the possible mechanism through which high insulin levels can trigger myopia. Experiments performed in chickens revealed that intravitreal insulin injection inhibited choroid thickening, elongated the anterior chamber, and thickened the lens, accelerating lens-induced axial myopia and inhibiting lens-induced hyperopia [46, 47]. Moreover, a higher total fibre intake was linked to a lower intake of total calories, including saturated fat, which is a known contributing factor to insulin resistance and an insulin antagonist [48, 49]. Laurence et al. [11] have reported that a higher saturated fat and cholesterol intake was associated with a longer axial length. This was similarly reported by Gardiner, who compared the diet of 33 active myopic and 251 stable myopic individuals and showed increased consumption of lipids and carbohydrates in the active group, suggesting the involvement of diet in the pathophysiology of myopia [50]. These findings support the hyperinsulinemia theory proposed by Cordain et al. [9].

A plausible alternative hypothesis is that dietary supplements with WG could prevent the development and progression of myopia as they contain calcium, iron, magnesium, manganese, copper, and zinc. These dietary supplements play an essential role in antioxidative processes and the biochemical remodelling of the sclera [51–54], which is important in facilitating the increase in axial length that results in myopia [55]. Huibi et al. have also revealed that zinc supplements in animal diets lead to the upregulation of copper–zinc superoxide dismutase, thereby inhibiting axial eyeball elongation [51].

Moreover, our study found that WGs remained the main determinants when analyses were stratified by sex. However, Berticat et al. [10] discovered that refined carbohydrate consumption is associated with myopia in children aged 5–19 years in a French paediatric population, with increased odds for girls and reduced odds for boys, possibly since chronic hyperglycemia is not commonly observed among boys due to their higher physical activity at all ages [10]. This study observed that intake > 50% of WGs was identified as a protective factor against myopia for boys and girls. However, a different relationship between WGs and myopia in the two groups (6–8years vs. 9–12 years) was observed, wherein WG intake was non-significant in children aged 6–8 years, while WG intake > 50% remained protective against myopia in children aged 9–12 years. This is likely due to the increased intensive near-work and educational pressures and less outdoor activities in older children. This also suggests that the dietary hypothesis cannot be tested independently, since the type and quantity of diet are associated with physical activities, which may be negatively correlated with time spent in near-work and outdoors [56, 57]. In this study, a multivariable analysis of intake of WGs in relation to myopia showed that WG intake > 50% remained protective against myopia after multivariable adjustment for time spent in near-work and outdoors. Finally, we also hypothesise that diets in cities or higher socioeconomic status families are more likely to be deficient in WG. As children from these backgrounds are likely to be myopic due to factors other than diet, this suggests a non-causal association of WG consumption with myopia, which could make the idea of dietary supplementation implausible. Further studies need to consider the socio-demographic risk factors to assess this possibility and investigate the underlying mechanisms by which WGs can affect myopia.

As mentioned, dietary intake is complex and highly variable. Factors such as environmental exposure and dietary assessment in this study may not be robust, as WGs are diverse, and we only assessed late-life diet. Additionally, because the dietary data were self-reported and collected retrospectively, subjecting it to recall errors, the information on WGs may not be accurate. Therefore, these findings should be replicated in other populations and compared with other dietary assessment methods.

Details on diet and an extensive list of covariates were collected using a questionnaire. Although this has obvious limitations, the results are still credible to some extent. First, our questionnaire was designed considering questionnaires developed and used successfully in other studies [36, 58, 59] and experts approved the final questionnaire. Second, the questionnaire was independently used by members of our research group with several years of experience in preventing and controlling myopia. To explore the relationship between WG and myopia, factors related to myopia were included as covariates to adjust for potential confounding effects. Last, if the same answer items were more than 70% for all the questionnaire items, the questionnaire was considered invalid, thus ensuring the credibility of our results.

## Conclusion

A clear inverse association was observed between the prevalence of myopia and WG intake. A higher proportion of WG intake had a protective effect against myopia development. Interestingly, modifying the form of grains consumed (whole vs. refined) could be a potential public health measure against myopia. As this study was exploratory, the data supporting this hypothesis are limited and it is not easy to provide strong evidence to establish accurate links between benefits of whole grain consumption and myopia. Therefore, further studies are warranted to validate our findings and investigate the underlying mechanisms by which WGs can affect myopia.

## Data Availability

The datasets used and analysed during the current study are available from the corresponding author (rwei@tmu.edu.cn) upon reasonable request.

## Abbreviations

WGs: Whole grains
OR: Odds ratio
